# Predicting iron and folate deficiency anaemias from standard blood testing: the mechanism and implications for clinical medicine and public health in developing countries

**DOI:** 10.1186/1742-4682-3-34

**Published:** 2006-10-09

**Authors:** Alan E Dugdale

**Affiliations:** 1 Department of Paediatrics and Child Health, School of Medicine, University of Queensland, Q 4006, Australia

## Abstract

**Background:**

Developing countries have high prevalence of diseases, but facilities to diagnose and treat them are limited. We must use available resources in ways not needed where there are sophisticated equipment and trained staff. Anaemia is common; iron deficiency affects health and productivity; folate deficiency in pregnant women causes foetal abnormalities. Few developing countries can measure serum folate or ferritin, but standard automated blood analyses are widely available and can help predict folate and iron deficiency. The RDW-CV% (coefficient of variation of the red cell width) measures the variability in the size of red blood cells (RBC) in routine automated analysis of blood cells, but is seldom reported. Levels of RDW-CV% and haemoglobin (Hb) can predict iron deficiency anaemia.

**Method and results:**

I have written a computer model based on the standard mechanism for blood formation and destruction. This shows that before anaemia develops and during recovery, there are both normal and abnormal RBC (small in iron deficiency and large in folate deficiency) in the circulation. The model calculates the abnormality in the RDW-CV% in standard automated blood analyses. In early iron deficiency and during recovery the full blood count shows the Hb near the lower limit of normal, a low MCV and a high RDW-CV%. A similar pattern, but with a higher MCV, develops in folate deficiency. Folate deficiency is often brief and may not cause anaemia. The high RDW-CV% may persist for three months.

**Conclusion:**

This long footprint could be medically useful for detecting folate deficiency and so limiting foetal damage in individuals and communities. Few clinicians or public health workers know about RDW-CV%. Standard blood reports for clinical use should include the RDW-CV% and note the possible significance of abnormal values.

## Background

A recent paper [1] has confirmed the findings of earlier authors [2,3] that we can predict the onset of iron deficiency anaemia using output from automated blood analyzers. These papers show that the blood changes parallel the low levels of iron stores. The main indicators are haemoglobin level (Hb) near the lower limit of normal and a high level of anisocytosis measured by the coefficient of variation (%) of the red cell distribution width (RDW-CV%). No mechanism for this finding was proposed. I describe a computer simulation model that follows the formation and destruction of red blood cells (RBC). When standard assumptions about red cell formation and destruction are used, and with an adequate supply of iron, the output of the model corresponds to the findings in normal blood. When iron is deficient the hitherto unexplained changes appear. The model also explains why people with macrocytic anaemia may have normal folate level even though the changes in the blood are due to folate deficiency [4].

The mechanism of the model is as follows. When normal bone marrow has adequate raw materials and hormonal stimulus, it produces enough normal RBC to maintain circulating Hb levels. The mean values for circulating RBC are mean cell volume (MCV) 90 ± 10 and RDW-CV 12%. When there is insufficient iron for normal haemopoiesis, the marrow produces microcytes with MCV 60 ± 7. After the start of altered haemopoiesis, the circulation contains a mixture of normal cells and microcytes, so the RDW-CV% increases rapidly, well before the overall levels of MCV and Hb drop below the normal range. When folate is lacking, the marrow produces macrocytes; the mixture of macrocytes and normal RBC raises the RDW-CV% before the MCV and Hb become abnormal.

### The computer model

The model is a computer programme that follows the changes in the RBC contained in one cubic millimetre of blood. The model is based on a matrix, 120 columns wide; each column contains the number and properties of RBC formed in a single day. The model runs with intervals of one (simulated) day. The new cells formed are entered into Column 1 of the matrix. At each iteration (day), the cohort of cells is moved one column to the right: Column 1 -> Column 2, Column 10 -> Column 11, and so on. It is assumed that the RBC in Column 120 have been destroyed and lost to the circulation. In this simple version of the model, a life span of 120 days is assumed. I also take the lower level for normal of Hb as 110 g/l (the World Health Organisation's lower limit for normal Hb for adult women) and the upper limit of normal RDW-CV% as 15%.

The input data are (a) the number of cells formed, (b) the mean cell volume, (c) the standard deviation of the mean cell volume, (d) the mean cell haemoglobin, (e) the number of days during which these conditions apply. It is assumed that the type of haemopoiesis switches from one form to another, e.g. normal to iron-deficient, within one day, but this is not critical for the working of the model. This simple version of the model also assumes that all RBCs have a lifespan of 120 days.

At the start of the run the matrix is empty. To populate the matrix, the model is run for 120 days with normal values for each of the input parameters. At any iteration, the characteristics of the RBC contained in one cubic millimetre of blood can be shown. The output values are RBC count per cubic mm, haemoglobin g/l, mean cell volume (MCV), mean cell haemoglobin (MCH), mean cell haemoglobin concentration (%) (MCHC%) and the red-cell distribution width (RDW-CV%). Once the matrix is populated, the type of haemopoiesis can be changed and the effect on the RBC parameters shown.

## Methods and results

### Iron deficiency

Table 1 shows the effects of iron deficiency on the full blood count. The model is first run for 120 days with normal haemopoiesis to populate the matrix (RBC/day 40000, MCV 90, SD of MCV 10, MCH 30). Following this, the model runs for 30 days (Days 0 – 30) with normal haemopoiesis, then for 150 days (Days 30 – 180) with iron deficient haemopoiesis (RBC/day 30000, MCV 60, SD of MCV 7, MCH 18).

**Table 1:** The changes in standard haematological findings with iron-deficient haemopoiesis.

Total	*RBC	*MCV	*MCV	*MCH	RBC	Hb	Ht	MCH	MCHC%	MCV	RDW-
Days	/day	fL	SD	Pg	/10^6	g/L		pg		fL	Cv%

0	40000	90	10	30	4.8	144	36	30	40	90	11.2
10	40000	90	10	30	4.8	144	36	30	40	90	11.2
20	30000	60	7	18	4.8	144	36	30	40	90	11.2.
***30***	***30000***	***60***	***7***	***18***	***4.7***	***137***	***34.7***	***29.2***	***39.6***	***88.1***	***14.0***
***40***	***30000***	***60***	***7***	***18***	***4.6***	***130***	***33.3***	***28.4***	***39.3***	***86.1***	***16.3***
***50***	***30000***	***60***	***7***	***18***	***4.5***	***124***	***31.9***	***27.6***	***38.9***	***84.0***	***18.3***
***60***	***30000***	***60***	***7***	***18***	***4.4***	***117***	***30.5***	***26.7***	***38.6***	***81.8***	***19.9***
***70***	***30000***	***60***	***7***	***18***	***4.3***	***111***	***29.0***	***25.8***	***38.2***	***79.5***	***21.3***
***80***	***30000***	***60***	***7***	***18***	***4.2***	***104***	***27.6***	***24.9***	***37.9***	***77.1***	***22.4***
***90***	***30000***	***60***	***7***	***18***	***4.1***	***97***	***26.0***	***23.9***	***37.6***	***74.6***	***23.2***
***100***	***30000***	***60***	***7***	***18***	***4.0***	***91***	***24.5***	***22.8***	***37.3***	***72.0***	***23.5***
***110***	***30000***	***60***	***7***	***18***	***3.9***	***84***	***22.9***	***21.7***	***36.9***	***69.2***	***23.2***
***120***	***30000***	***60***	***7***	***18***	***3.8***	***78***	***21.3***	***20.5***	***36.6***	***66.3***	***21.9***
***130***	***30000***	***60***	***7***	***18***	***3.7***	***71***	***19.7***	***19.3***	***36.3***	***63.2***	***18.8***
***140***	***30000***	***60***	***7***	***18***	***3.6***	***64***	***18***	***18***	***36***	***60***	***11.7***
***150***	***30000***	***60***	***7***	***18***	***3.6***	***64***	***18***	***18***	***36***	***60***	***11.7***
***160***	***30000***	***60***	***7***	***18***	***3.6***	***64***	***18***	***18***	***36***	***60***	***11.7***

The rows in normal print show the levels with normal haemopoiesis; the rows in bold print show the changes in levels during iron-deficiency. The columns marked with a * show the characteristics of the RBC formed on that day. The seven columns on the right show the haematological findings in the blood as would be found in a standard haematological report.

The MCV, MCH and RDW-CV% in the blood volume are initially normal. By the end of 30 days of abnormal haemopoiesis, the Hb and the MCV have decreased but remain within normal limits. However, the RDW-CV% becomes abnormally high, going from 11.2% to 18.3%, because of the mixture of circulating microcytes and normocytes. After 50 days of iron deficiency the Hb is 111, still within the normal range, but the RDW-CV% has risen further to 21.3%. The Hb continues to fall and the RDW-CV% continues to rise until all the normal cells formed before the iron-deficient haemopoiesis have been removed from the circulation. After this, there is a uniform population of iron deficient RBC, the Hb level stabilizes and the RDW-CV% returns to normal levels. The critical finding is that the RDW-CV% becomes abnormal while the Hb and MCV are still within normal range. This explains the findings [2,3] that a high RDW-CV% predicts later iron deficiency anaemia. When iron therapy is given and normal haemopoiesis returns (not shown here), there will again be both normal and iron-deficient RBC in the circulation so the RDW-CV% will again rise to abnormal levels until the microcytes formed during the period of iron deficiency reach the end of their lifespan.

### Folate deficiency

The body has extensive stores of iron, so iron deficiency is likely to be a long-term event producing the typical anaemia. Folate is a water-soluble vitamin. Body stores are relatively small and labile so temporary reduction of dietary intake can produce short-term (less than 1 month) folate deficiency, which is relieved by a few meals of folate-containing foods. Vegetable and fruit are the usual sources of folate; in developing countries, seasonal shortages often occur; in western countries, poor people may forgo these foods when less cash is available from welfare or other payments. Usually this would have little effect on health, but if the woman is in the first trimester of pregnancy, even a temporary deficiency of folate could produce severe and permanent effects on the foetus. The model shows that short-term deficiency produces characteristic effects on the RBC parameters.

Table 2 shows the changes associated with short-term folate deficiency. As in Table 1, the model is populated with normal RBC and then run for another 30 days with normal haempoiesis. From Day 10 to Day 40, haemopoiesis shifts from normal to the macrocytic pattern of folate deficiency. From Day 40 onwards, the folate deficiency has been corrected and haemopoiesis returns to the normal mode. During this short period of folate deficiency, the Hb drops and the MCV rises, but both remain within normal range. However, the RDW-CV% rapidly rises beyond the normal range. It remains high for more than 100 days after the end of the folate deficiency, that is until the cohort of macrocytes has left the circulation 120 days after the folate level has returned to normal.

**Table 2:** The changes in standard haematological findings with temporary folate-deficient haemopoiesis.

Day	*RBC	*MCV	*MCV	*MCH	RBC	Hb	Ht	MCH	MCHC	MCV	RDW
Tot	/day		SD		/c mm						CV%

0	40000	90	10	30	4.8	144	36	30	40	90	11.2
***10***	***20000***	***140***	***14***	***20***	***4.6***	***136***	***36.1***	***29.6***	***37.7***	***92.2***	***15.7***
***20***	***20000***	***140***	***14***	***20***	***4.4***	***128***	***36.1***	***29.1***	***35.5***	***94.5***	***18.8***
***30***	***20000***	***140***	***14***	***20***	***4.2***	***120***	***36.1***	***28.6***	***33.3***	***97.1***	***21.1***
40	40000	90	10	30	4.2	120	36.1	28.6	33.3	97.1	21.1
50	40000	90	10	30	4.2	120	36.1	28.6	33.3	97.1	21.1
60	40000	90	10	30	4.2	120	36.1	28.6	33.3	97.1	21.1
70	40000	90	10	30	4.2	120	36.1	28.6	33.3	97.1	21.1
80	40000	90	10	30	4.2	120	36.1	28.6	33.3	97.1	21.1
90	40000	90	10	30	4.2	120	36.1	28.6	33.3	97.1	21.1
100	40000	90	10	30	4.2	120	36.1	28.6	33.3	97.1	21.1
110	40000	90	10	30	4.2	120	36.1	28.6	33.3	97.1	21.1
120	40000	90	10	30	4.4	120	36.1	28.6	33.3	97.1	21.1
130	40000	90	10	30	4.6	128	36.1	29.1	35.5	94.5	18.8
140	40000	90	10	30	4.8	136	36.1	30	37.7	92.2	15.7
150	40000	90	10	30	4.8	144	36	30	40	90	11.4

The rows in normal print show the levels with normal haemopoiesis, the rows in bold print show the changes in levels during folate-deficiency.

## Discussion

This model puts numerical values to the well-known process of RBC formation and destruction. In so doing it shows the cause for hitherto unexplained observations [1-3] and predicts other clinical applications for routine blood analysis. The model shows that in iron deficiency, which could be due to inadequate iron stores or unavailability of iron resulting from acute infection, there is an early and prolonged rise in the RDW-CV% before the other parameters indicate anaemia. The RDW-CV% remains high until the blood is populated entirely by hypochromic cells. When iron is given and the haemopoiesis returns to normal, there is again an increase in the RDW-CV% (not shown in Table 1) for as long as there is a mixed population of normal and microcytic RBC in the circulation.

The model shows similar findings in short-term folate deficiency. The MCV increases and Hb decreases, but these remain within normal limits, while the RDW-CV% rises rapidly beyond the normal range. Folate levels can quickly return to normal when folate is fed, but the haematological effects remain for several months after the return of normal haemopoiesis until the macrocytes leave the circulation. This explains the lack of correlation between serum folate levels and macrocytic anemia [4,5]).

This method of detecting early iron and folate deficiencies is designed for use in countries where iron deficiency is very common – up to 50% in women of child-bearing age – folate deficiency much less common and other causes, with the exception of malaria and thalassaemia in some countries, uncommon. The model suggests that measures of MCV and RDW-CV% have two functions: first, to detect the possibility of a problem; second, to determine the nature of the problem. Uchida [2] reported a sensitivity of 77.1% for iron deficiency anaemia, 49.2% for iron deficiency anaemia plus latent iron deficiency, and specificity 90.6%. In thalassaemia minor, the RDW-CV% is more likely to be normal [6], but Green [7] stated that the discrimination is not good and other parameters that are measured by automated analysers but not reported, such as the cell haemoglobin distribution, may be better. In the anaemia of thalassaemia, the haemoglobin level is low but the RDW-CV% is usually normal [8]. In the anaemia if chronic illness the RDW-CV% is often within the normal range [9] and in malaria the RDW-CV% is low [10], but the best discriminator is a low platelet count [11].

This method cannot distinguish between folate and Vitamin B12 deficiencies, but B12 deficiency is much less common in most developing counties. When more than one of these essential nutrients is low, then the measurements of Hb, MCV and RDW-CV% reflect only the effects of the limiting nutrient. It is most unlikely that more than one of these will be a limiting nutrient at the same time. For example, if iron and folate levels are both low but iron is limiting nutrient, then the RBC will be small and hypochromic, with a low MCV and Hb and a high RDW-CV%. If iron is given to correct the iron deficiency, then there will be a partial response until haemopoiesis is limited by the low folate. At this time the MCV and RDW-CV% will show the folate deficiency.

The sensitivity and specificity of this method of predicting iron deficiency cannot be calculated from the computer model but are given in the papers cited. The sensitivity and specificity for folate deficiency cannot be determined. If there is long-term folate deficiency, serum folate levels will correspond with the haematological changes, but Robinson and Mladonovic [4] note that serum folate levels may be normal even in the presence of macrocytic anaemia. For short-term folate deficiencies that do not lead to anaemia but may cause foetal damage, the changes in MCV and RDW-CV% remain long after the folate level has returned to normal. There may be no other method for detecting recent folate deficiency and hence no gold standard to calculate sensitivity and specificity.

This is a very simple model designed to show the causes of hitherto unexplained changes in the MCV and RDW-CV% and their clinical significance. For simplicity, I have assumed that RBC formation is either normal or abnormal (iron deficient or folate deficient) although the model changes little if we assume a gradual transition. I have made no attempt to include the effects of other factors such as erythropoietin on blood formation. This method of detecting deficiencies in iron and folate does not supplant standard measures of serum iron and folate, but rather provides a useful tool in regions where anaemia is prevalent and resources limited.

### Technical description of the model

The model is written in DOS BASIC language and will run on any PC-based computer. A compiled version and the source code are both available. Details and code of the computer model are available from the author.

## Abbreviations

Hb Haemoglobin level (g/l)

RDW-CV% Coefficient of variation of the red cell width (%)

MCV Mean Red cell Volume (fl)

MCHC% Mean Cell Haemoglobin Concentration (%)

Ht Haematocrit

MCH Mean Cell Haemoglobin (pg)

## Competing interests

The author(s) declare that they have no competing interests.

## Authors' contributions

The author elucidated the mechanism described, designed and wrote the computer program and also wrote the paper.

**Figure 1:**
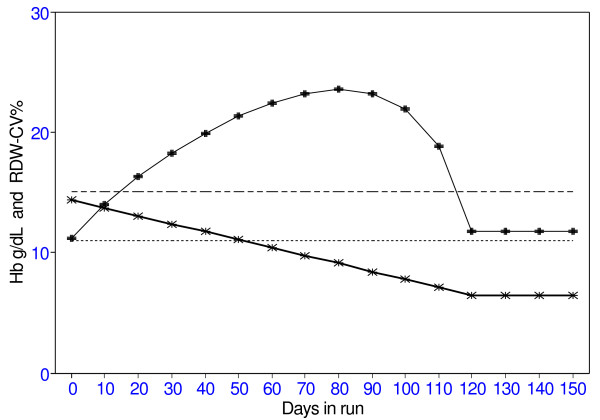
Changes in Hb and RDW-CV% with iron deficiency. RDW-CV% indicated by -+- Hb indicated by -x-

**Figure 2:**
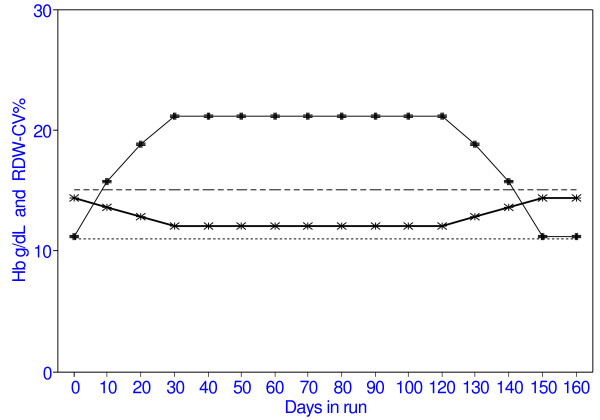
Changes in Hb and RDW-CV% with folate deficiency. RDW-CV% indicated by -+- Hb indicated by -x-
